# Magnesium Sulfate in Exacerbations of COPD in Patients Admitted to Internal Medicine Ward

**Published:** 2014

**Authors:** Mehrdad Solooki, Mirmohamad Miri, Majid Mokhtari, Morteza Valai, Mohammad Sistanizad, Mehran Kouchek

**Affiliations:** a *Department of Pulmonary and Critical Care Medicine, Imam Hossein Hospital, Shahid Beheshti University of Medical Sciences, Tehran, Iran.*; b *Department of Internal Medicine, Imam Hossein Hospital, Shahid Beheshti University of Medical Sciences, Tehran, Iran. *; c *Department of Clinical Pharmacy, Faculty of Pharmacy, Shahid Beheshti University of Medical Sciences, Tehran, Iran.*

**Keywords:** Magnesium sulfate, COPD, COPD exacerbation, Bronchodilatation

## Abstract

The purpose of this study was to examine the effect of intravenous magnesium sulfate on patients with COPD exacerbation requiring hospitalization.

In this randomized clinical trial 30 patients with COPD exacerbation were studied. Patients were randomly assigned to group A (case) who concurrent with standard therapy received 2 g magnesium sulfate in normal saline infused in 20 minutes on days one to three and group B (control) who received standard medications and placebo. PEFR and FEV1 were measured by before, 45 minutes and third day of entering the study. Vital signs HR, BP, RR, temperature and SpO2 were monitored during hospitalization.

21 males and 9 females patients with mean age of 68 ± 9 years, case 67 ± 10 and control 70 ± 8 were studied (15 patients in each arm of study). The mean pretreatment FEV1 was 26% ± 12, and 35% ± 18 in case and control groups respectively (P=0.137). FEV1 after 45 minutes in case group was 27% ± 9 and control group 36% ± 20 (p=0.122). FEV1 after 3 days of study was 32% ± 17 in case and 41% ± 22 in control groups (P=0.205). The mean pretreatment PEFR was 126 ± 76 l/min in case and 142 ± 62 l/min in control groups (P=0.46). Changes in PEFR were not significant 45 min (p=0.540) and 3 days (p=0.733) of the administration of intravenous magnesium sulfate. Duration of hospital stay between the two groups did not show any significant difference.

This study showed that administration of intravenous magnesium sulfate in hospitalized patients with COPD exacerbation neither revealed any significant bronchodilating effect nor reduced duration of hospital stay.

## Introduction

Chronic obstructive pulmonary disease (COPD) exacerbation is the one of the leading causes of morbidity, mortality, hospital admissions and increased healthcare utilization in modern medicine (1). 

The worldwide increase in COPD prevalence renders disease exacerbation an increasingly worrying phenomenon for clinicians, patients, healthcare organizations, and society in general. As a result, there is a mounting interest not only in designing optimal COPD treatment approaches but also in preventing its exacerbations (1-5). These realities emphasize the pressing need to improve treatment modalities for COPD exacerbations. 

Pulmonary rehabilitation, oxygen therapy, bronchodilators (β*2*-agonists and anticholinergic agents), inhaled and systemic corticosteroids and in critical situations mechanical ventilation are common treatments approaches in COPD (6, 7). However the need always exists to design new modalities and approaches to alleviate symptoms more effectively and decrease the frequency and severity of exacerbations. 

Intravenous magnesium sulfate has been known for its bronchodilating effect (8-10). The possible mechanism(s) of action of MgSO_4_ in offering benefit in COPD exacerbations may be calcium antagonism via calcium channel and counteraction of calcium-mediated smooth muscle contraction (11, 12). In addition early administration of intravenous magnesium sulfate in emergency department may reduce hospital admission rate (13). However, studies investigating the use of this agent in COPD exacerbations are scarce and inconclusive (14-16). 

We conducted this study to examine the effects of intravenous magnesium sulfate on respiratory functions (FEV1 and PEFR) of patients with COPD exacerbations in ED and during hospital stay.

*Patients and material*

We designed this prospective randomized-control double blind study at Imam Hussein Hospital affiliated to Shahid Beheshti University of Medical Sciences which is a large multispecialty medical center in Eastern Tehran caring for a wide range of medical, surgical and trauma related pathologies. Patients presenting with COPD exacerbation to emergency department were recruited for this study. ED management included bronchodilators, oxygen and corticosteroid. After 6 hours of ED management if there were no significant clinical improvement patients were admitted to internal medicine ward (pulmonary service). 

We included patients 40 years or older with COPD exacerbation. We excluded patients with contraindication for use of IV magnesium sulfate, patients unable to perform spirometry, presence of pneumonia, oral temperatures of 38 °C or more and systolic blood pressure less than 100 mmHg.

Upon admission to the floor creatinine, magnesium and ECG were recorded in all patients and treatment with oxygen for appropriate SpO_2_, bronchodilators such as Salbutamol 2 puffs every 6 hours, Ipratropium bromide 2 puffs every 6 hours, Methylprednisolone 60 mg slow intravenous infusion every 12 hours, and Azithromycin 500 mg first day then 250 mg/day for 4 days were administered. Stable patients with normal creatinine, serum magnesium levels and electrocardiograms were included in the study. 

Study detail was described to participating patients and informed written consents were obtained. Study protocol was approved by the institutional review board of Shahid Beheshti Medical University. 

Patients were randomly divided into group A (case) where, concurrent with standard treatment, 2 g magnesium sulfate diluted in 100 ml normal saline infused over 20 min was administered. In group B (control) patients received placebo of 100 cc normal saline and standard treatment. At the beginning of the study we measured peak expiratory flow rate (PEFR) and forced expiratory volume in 1 second (FEV_1_) using spirometer (Spiro analyzer ST-250 Fukuda Sangyo). PEFR and FEV_1_ were measured 45 minutes, second day and third day after entering the study. Vital signs HR, BP, RR, and temperature SpO_2_ were recorded in all patients.

The data were analyzed using Statistical Package for Social Studies version 17.0 (SPSS Inc. Chicago, Ill). Data were expressed as mean ± SD to compare within and between-groups differences were examined using t-test and chi square test. p-values <0.05 was considered significant.

## Results

Data from thirty patients, 21 males and 9 females, were suitable for final analysis (15 patients in each arm of study). Male to female ratio in case group was 11:4 and in control group was 10:5 (P=0.99). Mean age of the patients was 68 ± 9 years, in case group being 67 ± 10 years and in control 70 ± 8 years (P=0.34). The mean pretreatment FEV1 was 30% ± 16 in all patients, 26% ± 12 in case and 35% ± 18 in control group (P=0.137). FEV1 after 45 minutes in case group was 27% ± 9 and 36%±20 in control group (p=0.122) and after 3 days it was 32% ± 17 in case and 41 ± 22% in control group (P=0.205). The mean pretreatment PEFR was 134 ± 69 l/min in all patients, 126 ± 76 l/min in case and 142 ± 62 l/min in control group (P=0.46). After 45 min in case and control groups PEFR were 130±72 l/min and 145 ± 57 l/min respectively (p=0.540) and after 3 days it was 139 ± 79 l/min and 149 ± 79 l/min in case and control groups respectively (p=0.733) (Tables 1 and 2).

Duration of hospital stay between the two groups did not show any significant difference.

**Table 1:** Patients characteristics in case and control groups

		**Case **	**Control **	**p-value**
Age (year)		67 ± 10	70 ± 8	0.34
Sex (male/female)		11/4	10/5	0.99
Severity (GOLD criteria)				
	I	0	0	0.187
	II	0	4(27%)	
	III	5(33%)	4(27%)	
	IV	10(67%)	7(47%)	
Weight (Kg)		70±15	62±11	0.12
Height (cm)		166±6	164±4	0.46
Tobacco (pack year)		23 ± 18	22 ± 18	0.90
Tobacco>20 pack year		6(40%)	7 (47%)	0.75
History of admission		9(60%)	10(67%)	0.73
Baseline PEFR L/min		126 ± 76	142 ± 62	0.64
Baseline FEV1 (% of predicted)		26 ± 12	35 ± 18	0.13
Baseline SPO2%		89 ± 2	89 ± 1	0.82
Creatinine		1.17 ± 0.30	1.15 ± 0.26	0.85
Mg		2.11 ± 0.28	2.05 ± 0.40	0.68

**Table 2:** Pulmonary function measurements pre and post treatment in case and control groups

		**Case, mean±SD**	**Control, mean±SD**	**Difference (95% CI)**	**p-value**
PEFR (L/min)	Baseline	126 ± 76	142±62	-16(-68 to 36)	0.533
	45min	130 ± 72	145 ± 57	-15(-63 to 34)	0.54
	Change*	7 ± 19	5 ± 17		
	Day 3	139 ± 79	149 ± 74	-10(-67 to 47)	0.733
	Change*	17 ± 29	5 ± 19		
FEV1 (% 0f predicted)	Baseline	26 ± 12	35 ± 18	-9(-20 to 2)	0.117
	45min	27 ± 9	36 ± 20	-9(-21 to 3)	0.122
	Change*	14 ± 41	4 ± 33		
	Day3	32 ± 17	41 ± 22	-9(-24 to 5)	0.205
	Change*	31 ± 62	31 ± 72		
SPO2%	Pre	89 ± 2	89 ± 1	0(-1 to 1)	0.82
	Day 3	90 ± 2	90 ± 2	0(-2 to 1)	0.701
	Change*	2 ± 2	2 ± 2		

## Discussion

To evaluate the effectiveness of magnesium sulfate in COPD exacerbation we conducted this double blind randomized placebo-controlled clinical trial at our medical center. There were not any significant differences between case and control groups regarding age, sex, pretreatment FEV1 and PEFR. 

Following the administration of 2 g intravenous magnesium sulfate in treatment group and normal saline as placebo in control group, added to their standard COPD exacerbation therapy, we did not detect any significant differences in FEV1, PEFR and oxygen saturation.

Duration of hospital stay between the two groups did not show any significant difference. 

Therefore our results did not indicate magnesium sulfate to have significant bronchodilating effect reflecting on spirometric values (PEFR and FEV_1_) measured in our study patients with COPD exacerbation. 

Our results were in line with González *et al*. 2006 placebo controlled randomized trial from Spain which did not show intravenous magnesium sulfate to have significant bronchodilating effect in COPD exacerbations. (14) However these results are in contrast with Skorodin *et al.* study which reported bronchodilating effect of magnesium sulfate administration in these patients. This effect was more prominent than inhaled β*2*-agonists alone (15). In another study Amaral *et al.* in 2008 which reported IV magnesium sulfate administration in stable COPD patients could decrease lung hyperinflation and improve respiratory muscle strength (16). 

The difference between our results and Skorodin and Amaral studies was possibly due to differences in methodologies where they measured the level of bronchial obstruction using either PEFR or FEV_1_ as it might have underestimated the extent of bronchial obstruction compared to our study where we measured both PEFR and FEV_1_ by conventional spirometry. 

Nannini *et al*., in another study, claimed that administration of inhaled isotonic magnesium sulfate simultaneous with salbutamol had better bronchodilating effect. (17) Similarly González hypothesized that intravenous magnesium sulfate in patients with exacerbations of COPD improved the bronchodilating effect of inhaled β*2*-agonists. (14) We cannot compare our results with these findings as we did not evaluate bronchodilating effect of magnesium sulfate along with other bronchodilators agents. 

The factors such as relatively small sample size, short duration of follow-up, absence of respiratory and general symptoms questionnaire are some of the limitations of this study.

Larger investigations with longer follow up time are required to address the role of magnesium sulfate in COPD exacerbation.

## Conclusion

In conclusion our findings in this study indicated that the administration of intravenous magnesium sulfate in hospitalized patients with COPD exacerbation did not demonstrate bronchodilating effect nor did it shorten the duration of hospital stay.
